# Barley farmland harbors a highly homogeneous soil bacterial community compared to wild ecosystems in the Qinghai-Xizang Plateau

**DOI:** 10.3389/fmicb.2024.1418161

**Published:** 2024-06-24

**Authors:** Xiaolin Wang, Yibin Yang, Qiong Nan, Jian-Wei Guo, Zhiyuan Tan, Xiaoming Shao, Changfu Tian

**Affiliations:** ^1^Guangdong Provincial Key Laboratory of Plant Molecular Breeding, South China Agricultural University, Guangzhou, China; ^2^State Key Laboratory of Agrobiotechnology, College of Biological Sciences, China Agricultural University, Beijing, China; ^3^Institute of Environmental Science and Technology, College of Environment and Resource Science, Zhejiang University, Hangzhou, China; ^4^College of Agronomy and Life Sciences, Yunnan Urban Agricultural Engineering and Technological Research Center, Kunming University, Kunming, China

**Keywords:** microbial biogeography, *Acidobacteria*, pH, Xizang, highland barley

## Abstract

**Introduction:**

Understanding patterns and processes of microbial biogeography in soils is important for monitoring ecological responses to human activities, particularly in ecologically vulnerable areas such as the Qinghai-Xizang Plateau. Highland barley is the staple food of local people and has mainly been cultivated along the Yarlung Zangbo River valley in Xizang.

**Methods:**

Here we investigated soil bacterial communities from 33 sampling sites of highland barley farmland in this region and compared them to those from wild ecosystems including alpine tundra, meadow, forest, and swamp. Additionally, the effects of environmental factors on bacterial communities, as well as the relative importance of stochastic and deterministic processes in shaping the beta diversity of soil bacterial communities in alpine ecosystems were assessed.

**Results:**

In contrast to soils of wild ecosystems, these farmland samples harbored a highly homogeneous bacterial community without significant correlations with geographic, elevation, and edaphic distances. Discriminant bacterial taxa identified for farmland samples belong to *Acidobacteria*, with *Acidobacteria Gp4* as the dominant clade. Although *Acidobacteria* were the most abundant members in all ecosystems, characterized bacterial taxa of meadow and forest were members of other phyla such as *Proteobacteria* and *Verrucomicrobia*. pH and organic matter were major edaphic attributes shaping these observed patterns across ecosystems. Null model analyses revealed that the deterministic assembly was dominant in bacterial communities in highland barley farmland and tundra soils, whereas stochastic assembly also contributed a large fraction to the assembly of bacterial communities in forest, meadow and swamp soils.

**Discussion:**

These findings provide an insight into the consequences of human activities and agricultural intensification on taxonomic homogenization of soil bacterial communities in the Qinghai-Xizang Plateau.

## 1 Introduction

Microbial biogeography investigates the geographical variation of microorganisms across space and time, and uncovers the processes that cause this variation in microbial assemblage (Hanson et al., 2012; Xu et al., 2020; Wan et al., 2023). With the revolutionary advance in molecular phylogenetic approaches, sequencing techniques and computational methods in recent years (Kozich et al., 2013; Larin et al., 2024), extensive studies have successfully established the biogeographic patterns of bacteria across a broad spectrum of ecosystems (Nemergut et al., 2013). Now the major theme of this field has focused on identifying the mechanisms that shape these patterns (Hanson et al., 2012; Nemergut et al., 2013; Ruff et al., 2015; Cao et al., 2016; Kumar et al., 2017).

It has been acknowledged that edaphic variables, such as pH (Fierer and Jackson, 2006; Cao et al., 2016), salinity (Feng et al., 2023), and carbon availability (Fierer et al., 2007; Walsh et al., 2016), have a pivotal role in determining microbial biogeography. For example, it has been reported that the relative abundance of *Acidobacteria*, ubiquitous and abundant members of soil bacterial communities (Kielak et al., 2016; Delgado-Baquerizo et al., 2018), is negatively correlated with pH (Wang et al., 2023). These findings support a classic deterministic process of selection via abiotic features in determining microbial community assemblage. However, variation of microbial communities can also be affected by stochastic processes such as dispersal (Hawkins and Zeglin, 2022), evolutionary diversification (Shapiro et al., 2012), and ecological drift (Jones et al., 2023). Given the irreplaceable fundamental functions of microorganisms in biosphere, understanding both patterns and processes of microbial biogeography is not only helpful for us to investigate forces generating biodiversity, but also crucial for monitoring ecological responses to environmental changes caused by human activities.

Highland barley is the staple food for local people and an important livestock feed in the Qinghai-Xizang Plateau, which is one of the domestication centers for cultivated barley (Zeng et al., 2015). To improve the sustainable development of highland barley farmland in the ecologically vulnerable Qinghai-Xizang Plateau, it is important to know the characteristics of soil bacterial communities in the field. To this end, we firstly determined the beta diversity of soil bacterial communities in the highland barley farmland across 33 sampling sites along the Yarlung Zangbo River valley in the Qinghai-Xizang Plateau. The identified biogeographical pattern of soil bacterial communities and processes shaping it were compared to those of 33 soil samples in wild ecosystems (including tundra, swamp, meadow and forest), which are of different levels of ecological fragility.

## 2 Materials and methods

### 2.1 Sampling, DNA extraction, soil physicochemical properties characterization

**Figure 1:**
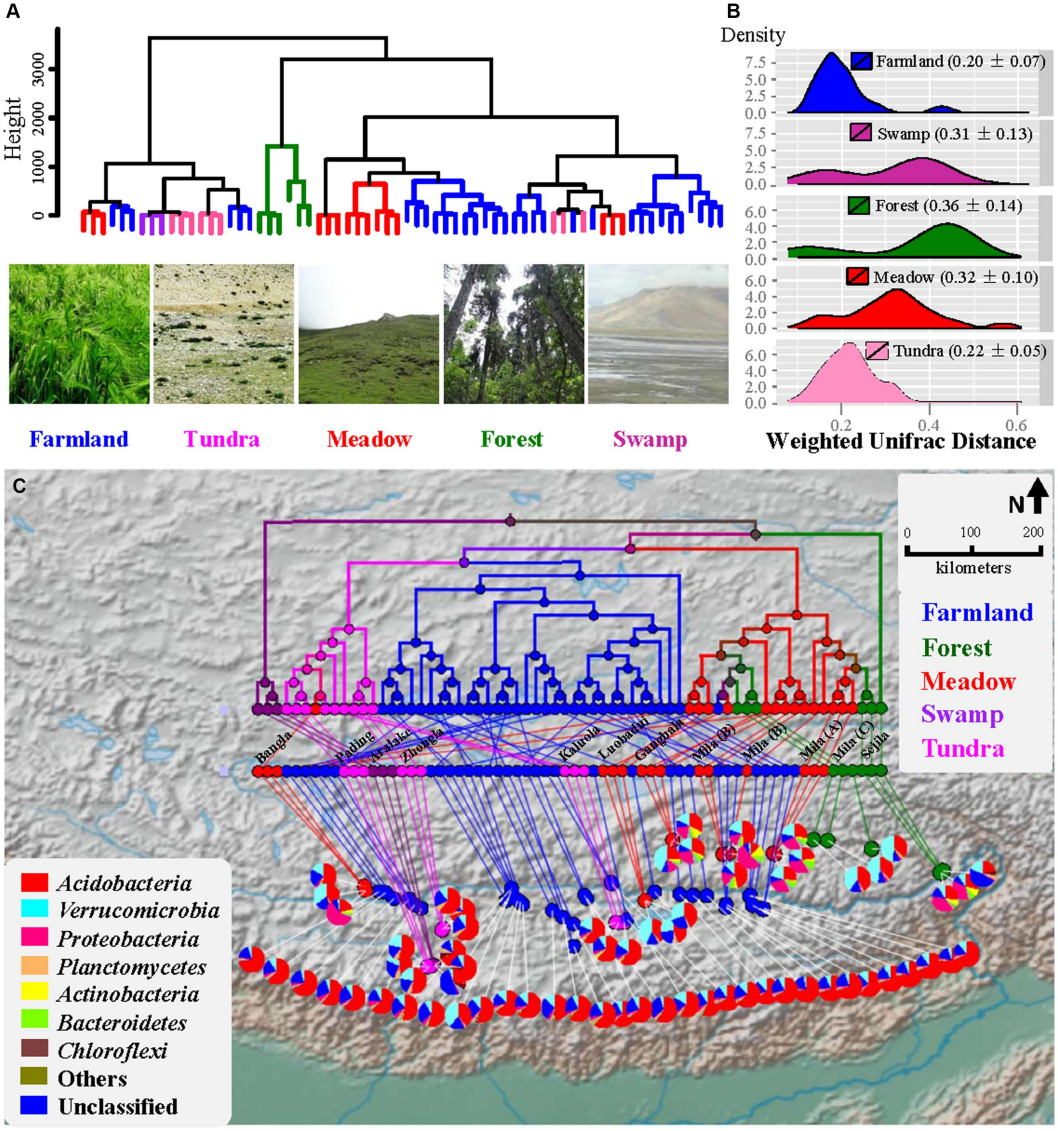
Beta diversity analysis of bacterial community. **(A)** UPGMA dendrogram based on geographic distance. Photos show plants of Xizang Highland barley, and the landscape of alpine tundra and meadow. **(B)** Density plot showing the distribution of weighted UniFrac distance (mean ± SD). **(C)** Geospatial biodiversity mapping analysis comparing bacterial communities across Xizang biomes using weighted UniFrac distance. Branches are colored based on the ecosystems they represent. Pie charts represent relative abundance of bacteria taxa at phylum level for each site. Geographic origins of soil samples from wild ecosystems are shown.

Highland barley farmland soils were collected from 33 sampling sites in Xizang, China, with 12 samples collected along the Nyang Qu River (an upper tributary of Yarlung Zangbo River), 12 samples from the downstream of the Yarlung Zangbo River (where the Lhasa River joined), and nine samples from the midstream of the Yarlung Zangbo River (Supplementary Table S1 and Figure 1). Non-farmland soils were collected from 30 sites across eight alpine mountains (Mt. Bangla, Mt. Gangbala, Mt. Kaluola, Mt. Luobadui, Mt. Mila, Mt. Sejila, Mt. Pading and Mt. Zhongla) in the same region (with fifteen alpine meadow sites, nine alpine tundra sites, and six high-frigid forest sites), and three sites in Aralake swamp, a dry lakebed located in the Everest Wetland Nature Reserve (Supplementary Table S1 and Figure 1). Samples were collected during the growing season when the barley starts sprouting and the alpine plants flourishing and flowering. At each site, three areas (0.5 m x 0.5 m) were probed, with dominant vegetation recorded *in situ*. Soil samples (0–15 cm) from three areas were randomly collected and mixed as a single sample for each sampling site. Soil samples were stored in ice bags, transported to laboratory and preserved at −80°C before DNA extraction. Total DNA of soil samples were extracted using the Fast DNA™ SPIN Kit (MP biomedicals, Cleveland, OH, United States). The quality and quantity of DNA were checked with a NanoDrop device (ND-1000, Thermo Fisher, United States). Moreover, total N, available phosphorus (P), organic matter (OM), available potassium (K), electrical conductivity (EC), pH, and ion concentrations of Ca^2+^, Mg^2+^, Na^+^, Cl^−^, K^+^, HCO_3_^−^, and SO_4_^2−^ for air-dried soil samples were determined at the Plant Nutrient and Resource Research Institution, Beijing Academy of Agriculture and Forestry Sciences, China (Supplementary Table S1), using the methods described before (Wang et al., 2018). Non-parametric pairwise comparison (Dunn’s Post-Hoc test) between the 5 alpine ecotypes showed no significant difference based on the Euclidean distances of geographic (Supplementary Figure S1A) and elevation (Supplementary Figure S1B) characteristics, however, the edaphic distances of farmland are significantly lower than that of meadow and tundra (Supplementary Figure S1C, *p*-values = 9.85e−04 and 0.02, respectively). Climate data such as mean annual temperature (AMT) and annual precipitation (AP) of the sampling region were acquired from DIVA-GIS^1^ at the 2.5 arc-minutes resolution level. AMT and AP were then predicted and cross validated with linear model using latitude, longitude and altitude as explanatory variables in R before interpolating into sampling sites (Supplementary Table S1).

### 2.2 Library construction for 16S rRNA gene sequencing

PCR amplifications were performed on 66 DNA samples using the 515F/806R primer set (515F: GTGYCAGCMGCCGCGGTAA and 806R: GGACTACNVGGGTWTCTAAT), which targets the V4 region of the 16S rRNA gene (Caporaso et al., 2012). The PCR products with P5/P7 adapters and double indexes were purified with double volume of AMPure XP beads, and fluorescence quantified (Qubit 2.0 Fluorometer, ThermoFisher Scientific, United States) with Qubit dsDNA HS assay kit (Invitrogen, United States). Multiple samples were pooled together in equimolar concentrations. Then 10 ng of pooled PCR products were sequenced on the Illumina MiSeq platform (paired-end reads of 300 bp; Illumina, United States) at the National Human Genome Centre of China in Shanghai, China, according to the manufacturer’s manual.

### 2.3 Sequence processing and bacteria diversity analysis

Raw 16S rRNA gene fastaq files were processed using Mothur v.1.35.1 according to Miseq SOP (Kozich et al., 2013). Reads were firstly combined and screened to reduce sequencing errors, then aligned against the SILVA-based bacterial reference (Release 138.1). The aligned reads were further de-noised and chimeras were removed using the UCHIME algorithm (Edgar et al., 2011). Any sequences not belonging to bacterial 16S rRNA gene, such as homologs from archaea, chloroplasts, and mitochondria, were classified using the Bayesian classifier and removed before downstream analysis. The optimized sequences were then normalized to 4,052 sequences per sample (the smallest sequencing depth for tested samples) before calculating taxonomy-based diversity and phylogeny-based diversity using Mothur and FastTree, respectively, (Price et al., 2009; Schloss et al., 2009).

For taxonomy-based analysis, the quality-controlled subsample sequences were clustered into operational taxonomic units (OTUs) that share ≥97% sequence similarity. Rare OTUs constituting less than 0.001% of the total normalized filtered sequences were removed, for rare OTUs may result in inflated estimates of diversity (Bokulich et al., 2013). Taxonomic information was then assigned for the remaining OTUs. For phylogeny-based analysis, the subsample sequences with rare OTUs removed were analyzed using FastTree software to compute an approximately-maximum-likelihood phylogenetic tree (Price et al., 2009). The phylogenetic tree of the dominant clade of bacterial orders and their relative abundance across ecosystems were visualized in EvolView (Zhang et al., 2012).

Alpha diversity was analyzed using the number of OTUs observed, the inverse Simpson D (Simpson, 1949), as well as Faith’s phylogenetic diversity (PD) (Helmus et al., 2007) by randomly selecting 3,000 sequences per sample 1,000 times and calculating the average (Supplementary Table S2). Beta diversity was calculated either using the taxonomy-based Bray–Curtis dissimilarity metric or the phylogeny-based weighted UniFrac metric generated from the filtered sequences (evenly sampled at 3,000 reads per sample with 1,000 iterations). The resulting distances were further visualized by using GenGIS geographical mapping analysis (Parks et al., 2009). Analysis of similarity statistics (ANOSIM) (Clarke, 1993) and homogeneity of molecular variance (HOMOVA) (Stewart and Excoffier, 1996) were used to test significant differences in composition and variation of bacterial communities.

Discriminant bacteria from phylum to family level were identified for different ecosystems using linear discriminant analysis effect size (LEfSe) (Segata et al., 2011). Taxa with significant differential abundance (Kruskal–Wallis sum-rank test, *p*-value <0.05; LEfSe >4) were used to generate taxonomic cladograms illustrating differences between Xizang ecosystems. Indicator OTU analysis (Dufrene and Legendre, 1997) was used to identify potential specialist for each ecosystem at the OTU level. This approach can uncover OTU, which is present in most sites of a group and has a high relative abundance within that group compared to the other groups, with statistical significance evaluated using a randomization procedure. OTUs with IV > 0.5 (indicator value) and *p*-value <0.05 were classified as group specialists. The high abundant indicators (OTUs representing larger than 0.05% of the total filtered and normalized sequences) identified by this method were selected for species assemblages analysis using Kendall’s W coefficient of concordance (Legendre, 2005). An overall test of independence of all abundant indicator OTUs was first executed. If the null hypothesis was refused, correlated species were divided into groups and permutation tests were used to test the contribution of each OTU to the overall statistic within each group. OTUs representing larger than 0.05% of the total subsample sequences were used in the analysis to avoid inflated estimates of indicator OTUs caused by rare taxa.

### 2.4 Effects of environmental factors on bacterial communities

The variation in phylogenetic diversity was measured using null-model-based phylogenetic β-diversity metrics (βNTI) to classify community pairs into underlying drivers of deterministic and stochastic processes, as described in previous studies (Dini-Andreote et al., 2015; Jiao et al., 2022). Briefly, when βNTI is less than −2, it suggests a significantly lower phylogenetic turnover than expected under a null model of community assembly, indicating the presence of homogeneous selection, where similar species tend to coexist. Conversely, a βNTI greater than 2 indicates significantly higher phylogenetic turnover, suggesting variable selection, where dissimilar species coexist. If the absolute value of βNTI is less than 2, it indicates that stochastic processes, such as dispersal, drift, or speciation, play a dominant role in shaping the community structure. Mantel and Partial Mantel tests were performed based on weighted UniFrac distance and Euclidean distances (elevation, geographic, or edaphic distances) to study the correlation between environmental factors and soil bacterial communities. After removing highly correlated environment variables (Spearman *ρ* > 0.8), Hellinger transformed OTU abundance data were projected to environmental variables: geographic (G), elevation (A), edaphic attributes (E) for correlation analysis using redundancy analysis (RDA). The variance degree of bacterial taxa accounted by those three explanatory variables and their combined effects were further determined using the partitioning functions based on RDA analysis. These analyses were all carried out by using functions in the vegan package in R software (v 3.1.3 and v 3.2.2) (Legendre and Legendre, 2012; Oksanen et al., 2016). Partial correlation analysis was used to control the variation of other edaphic factors when the correlation between pH/OM and relative abundance of *Acidobacteria* was studied.

## 3 Results

### 3.1 Homogenous bacterial community in the barley field in contrast to other alpine ecosystems

From 66 soil samples of different ecosystems along the Yarlung Zangbo River valley (Figure 1A), 745,451 quality-trimmed sequences (an average of 253 bp) were generated with a reduction of 27.5% of total raw sequencing reads. In total, 5,329 OTUs were kept after removing rare OTUs and the number of observed OTUs per sample ranged from 352 to 779 (Supplementary Table S2). The highland barley farmland soils harbored the lowest variation in bacterial community structure as measured by using weighted UniFrac distance (Figure 1B and Supplementary Figure S1D) compared to those of forest (Dunn’s Post-Hoc test, *p*-values = 1.84e−04) and meadow (*p*-values = 3.72e−24), but showed no significant difference to those of tundra (*p*-values >0.05). HOMOVA (Table 1) showed that the amount of bacterial beta diversity within farmland samples was different from those of forest and meadow (all *p*-values <0.05), but not from those of tundra and swamp (all *p*-values >0.05). ANOSIM showed that the bacterial composition of farmland soils was different from those of wild ecosystems (all *p*-values <0.01; Table 1). Dissimilarity of bacterial composition between different wild ecosystems was also uncovered for 5/6 pairs (all *p*-values <0.05), except the meadow-forest pair (*p*-value >0.05).

**Table 1:** Analysis of similarities (ANOSIM) and homogeneity of molecular variance (HOMOVA) of bacterial beta-diversity.

	Bacteria Bray-Curtis				Bacterial weighted UniFrac			
	ANOSIM		HOMOVA		ANOSIM		HOMOVA	
Comparison	R	*p*-value	B	*p*-value	R	*p*-value	B	*p*-value
Farmland-swamp	0.94	0.001	0.60	0.081	0.99	<0.001	0.71	0.256
Farmland-forest	0.93	<0.001	1.17	0.030	0.92	<0.001	3.47	<0.001
Farmland-meadow	0.87	<0.001	2.52	<0.001	0.87	<0.001	3.73	0.003
Farmland-tundra	0.87	<0.001	0.28	0.225	0.77	<0.001	0.02	0.808
Tundra-swamp	0.99	0.006	0.16	0.329	1.00	0.004	0.45	0.372
Tundra-forest	0.64	<0.001	0.24	0.165	0.68	<0.001	1.71	0.007
Tundra-meadow	0.27	0.004	0.38	0.010	0.38	0.001	1.34	0.002
Meadow-swamp	0.98	0.001	0.00	0.906	0.96	0.001	0.00	0.999
Meadow-forest	0.14	0.150	0.00	0.993	0.17	0.057	0.16	0.286
Forest-swamp	1.00	0.008	0.00	0.858	0.99	0.012	0.06	0.192

The variation patterns of the soil bacterial community within or between ecosystems were further analyzed using geospatial biodiversity mapping based on the pairwise weighted UniFrac distance (Figure 1C). Generally, independent of geographical distance between samples (Figure 1A), farmland samples formed a homogeneous cluster, which is closer to the alpine tundra cluster than the intermingled forest-meadow branches. A significant distance decay of community similarity was revealed for samples of tundra (Supplementary Figure S2, R^2^ = 0.19, *p*-value <0.01) and meadow (R^2^ = 0.12, *p*-value <0.001), but not for farmland (R^2^ = 0.0056, *p*-value >0.05) and forest samples (R^2^ = 0.0526, *p*-value >0.05). Swamp samples were not analyzed herein due to the small sample size and their immediate vicinity (less than 10 meters’ distance). In short, soil bacterial communities in the highland barley farmland were highly homogenous along the Yarlung Zangbo River valley compared to those of wild ecosystems investigated herein in the same region.

### 3.2 The relative importance of stochastic and deterministic processes in shaping the beta diversity of soil bacterial communities in alpine ecosystems in Xizang

**Figure 2:**
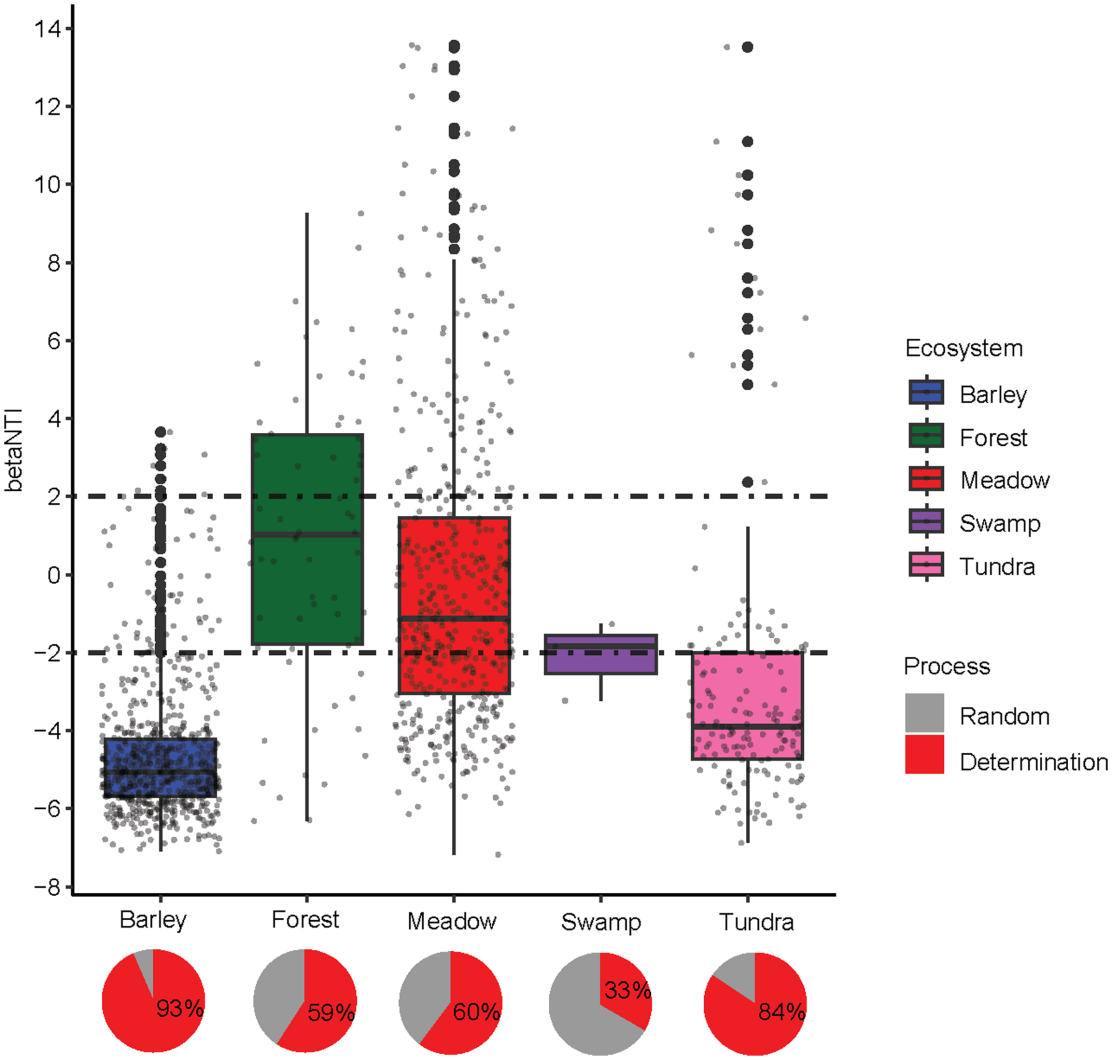
The distributions of between community analog (βNTI) across alpine ecosystems. The horizontal dashed lines indicate the βNTI values of +2 and − 2. Pie plots shows the relative contribution of each ecological process to community assembly.

Null model analyses revealed that the deterministic assembly was dominant in bacterial communities in highland barley farmland (93%) and tundra (84%) soils, whereas stochastic assembly also contributed a large fraction to the assembly of bacterial communities in forest (41%), meadow (40%) and swamp (67%) soils (Figure 2). In particular, the assembly of bacterial communities in highland barley farmland and tundra soil was governed by the homogeneous selection (βNTI < −2, Figure 2), in accordance with the homogenous bacterial community in these two ecosystems contrast to other alpine ecosystems. The relationships between phylogenetic diversity and major factors were also used to infer changes in the relative influences of deterministic and stochastic assembly processes, which was assessed using the Mantel tests that correlated the weighted UniFrac distance matrices with the Euclidean distance matrices of environmental variables. The highly correlated environmental variables (Spearman *ρ* > 0.8) among soil physicochemical properties, climatic and topographical features (Supplementary Table S1) were removed. The final variables used were as follows: latitude, longitude, altitude, organic matter, available phosphorus, available potassium, electrical conductivity, pH, Na^+^, Cl^−^ and SO_4_^2−^. All selected environmental features except K^+^ and SO_4_^2−^ exhibited significant differences between ecosystems (ANOVA, all *p*-values <0.01, Supplementary Table S3).

The Mantel and Partial Mantel tests revealed that the beta diversity, based on weighted UniFrac distance (Table 2), within farmland samples was not correlated with the distance matrix of geographic (latitude and longitude), elevation (altitude) or edaphic characteristics (all *p*-values >0.05), indicated that the deterministic assembly of bacterial communities of highland barley farmland could not be explained by measured environmental variables. By contrast, the beta diversity of tundra samples correlated with edaphic distance (*ρ* = 0.396, *p*-value <0.05) in the Mantel test, and after controlling for geographic (*ρ* = 0.359, *p*-value <0.05) and elevation distances (*ρ* = 0.397, *p*-value <0.05) in the Partial Mantel test, indicating significant effects of deterministic factors. Similarly, the beta diversity of meadow samples correlated with geographic (*ρ* = 0.366, *p*-value <0.01) and edaphic distances (*ρ* = 0.510, *p*-value <0.001) in the Mantel test. The beta diversity of forest samples showed no correlation with elevation, geographic and edaphic factors (Table 2, all *p*-values >0.05), despite its high level of similarity to that of meadow samples (Figure 1B).

**Table 2:** Mantel and partial Mantel tests for the correlation between Weighted UniFrac distances of soil bacterial communities and the Euclidean distances of all explanatory variables.

Weighted UniFrac		Ecosystems					All
Effects of	Factor controlled	Farmland	Tundra	Meadow	Forest	Swamp	All samples
Geographic		0.048	0.214	0.366^**^	0.3143	NA	0.464^***^
Elevation		−0.028	0	0.039	0.1107	NA	0.088^*^
Edaphic		0.120	0.396^*^	0.510^***^	−0.1036	NA	0.491^***^
Geographic	Edaphic	0.040	0.122	0.295^*^	0.4521	NA	0.381^***^
	Elevation	0.071	0.214	0.364^**^	0.3317	NA	0.458^***^
Elevation	Edaphic	−0.048	0.030	−0.061	0.1191	NA	0.074
	Geographic	−0.060	0.006	−0.001	0.1567	NA	0.019
Edaphic	Geographic	0.117	0.359^*^	0.469^**^	−0.356	NA	0.416^***^
	Elevation	0.126	0.397^*^	0.512^***^	−0.1125	NA	0.489^***^

Elevation, geographic and edaphic distances were analyzed herein using Spearman’s rho for different ecosystems and all samples. ^***^p-value < 0.001; ^**^p-value < 0.01; ^*^p-value < 0.05 (based on 10,000 permutations). NA, swamp samples are not analyzed due to the small sample size. All explanatory distances were measured as Euclidean distance using all variables standardized to have a mean of zero.

### 3.3 Discriminant bacterial taxa in alpine ecosystems

**Figure 3:**
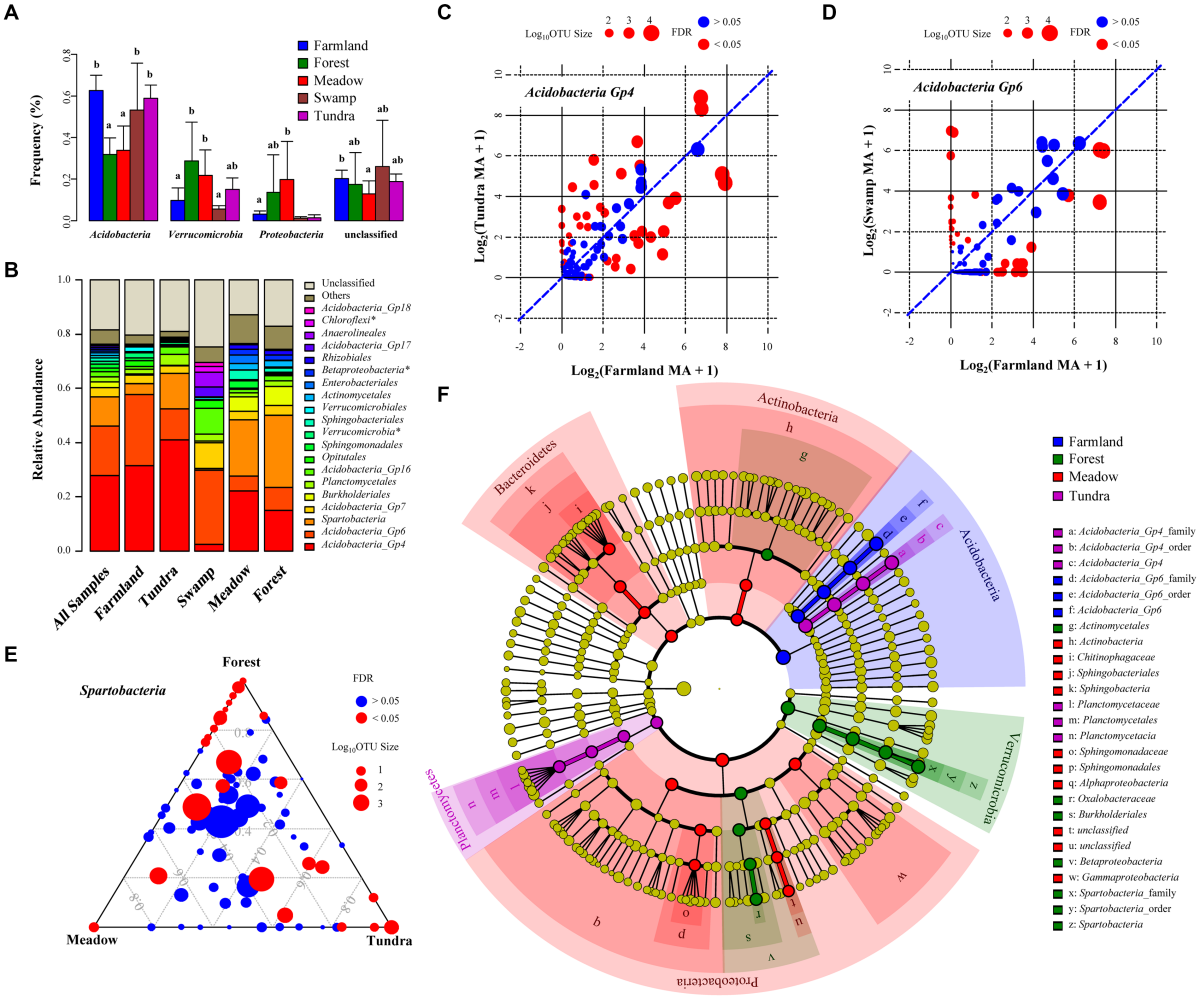
Discriminant bacterial taxa in alpine ecosystems. **(A)** Average relative abundance of major bacterial phyla. Error bars represent SD. Different letters indicate significant difference between ecosystems (*p*-value <0.05, Tukey’s test). **(B)** Relative abundance of the top 20 taxa at the order level. * represents the unclassified orders, for which phylum/class name is used. **(C-E)** Comparison of relative abundance of all OTUs classified as *Acidobacteria_Gp4* in the farmland and tundra groups **(C)**, *Acidobacteria_Gp6* in the farmland and swamp groups **(D)**, and *Spartobacteria* among mountain biomes **(E)**. OTUs represented by red points are those of significantly different abundance between groups (Kruskal-Wallis test, p-value <0.05). The points are sized according to their log10-transformed number of reads in two groups. The y = x blue dashed line indicates equal average relative abundance. **(F)** Indicator bacterial taxa identified by linear discriminant analysis effect size (LEfSe).

Among the 66 soil samples collected from different ecosystems, bacterial communities were dominated by the phyla *Acidobacteria* (52 ± 16%, average ± SD), *Verrucomicrobia* (15 ± 11%), *Proteobacteria* (7 ± 12%), *Planctomycetes* (2 ± 1%), *Bacteroidetes* (1 ± 2%), *Actinobacteria* (1 ± 3%), and *Chloroflexi* (1 ± 2%) (Figures 1C, 3A). Relative abundances of these seven phyla were different across the five ecosystems (multivariate analysis of variance, *p*-value <0.001). Relative abundances of *Acidobacteria* in soil samples of farmland, swamp and tundra were higher than those from forest and meadow (Figure 3A, Tukey’s test, *p*-value <0.05), whereas *Verrucomicrobia, Proteobacteria, Bacteroidetes* and *Actinobacteria* showed the opposite pattern.

Notably, *Acidobacteria_Gp4, Acidobacteria_Gp6* and *Spartobacteria* dominated bacterial community composition, from class to family level. These three clades accounted for 56.9% of the total sequence in all soil samples (Figure 3B). *Acidobacteria_Gp4* was 31.6 and 40.9% of the total sequences in farmland and tundra, respectively. *Acidobacteria_Gp6* accounted for 26.2 and 27.2% of the total sequences in farmland and swamp, respectively. *Spartobacteria* was 26.8, 20.8 and 13.1% of the overall sequences in forest, alpine meadow and tundra, respectively (Figure 3B). Although *Acidobacteria_Gp4* dominated in both farmland and tundra soils, one quarter of its OTUs (361 OTUs in total) showed biased distribution between these two ecosystems (Kruskal Wallis test with FDR-adjusted *p*-value <0.05; Figure 3C and Supplementary Figure S3). Similarly, 14.1% of OTUs belonging to *Acidobacteria_Gp6* (340 OTUs in total) were different in relative abundance between farmland and swamp (Figure 3D and Supplementary Figure S4), 27.7% of OTUs within *Spartobacteria* (139 OTUs in total) were different in relative abundance between forest, alpine meadow and tundra (Figure 3E and Supplementary Figure S5). These results imply the existence of potential discriminant bacterial taxa for each ecosystem.

LEfSe identified 37 significantly discriminant bacterial taxa for Xizang ecosystems (Kruskal–Wallis sum-rank test, *p*-value <0.05; LEfSe >4), such as *Acidobacteria_Gp6* clade (from phylum to family level) for the highland barley farmland, *Acidobacteria_Gp4* and *Planctomycetaceae* clades for tundra, *Oxalobacteraceae* and *Spartobacteria* clades in forest soils, *Sphingomonadaceae* and *Chitinophagaceae* clades in alpine meadow soils (Figure 3F). Swamp samples were not analyzed in LEfSe due to small sample size. Indicator species analysis identified 50 indicator OTUs with IV > 0.5 (indicator value) and *p*-value <0.05 (Supplementary Table S4 and Supplementary Figure S6). *Acidobacteria_Gp4* OTUs have more probability being identified as indicator taxa for farmland (5 OTUs) and tundra (4 OTUs), while *Proteobacteria* OTUs are more frequently identified as indicator taxa for forest and meadow. To elucidate potential relationship among these indicator OTUs, Kendall’s W coefficient of concordance was used to identify local assemblages of the indicator OTUs and clustered the 50 indicator OTUs into three assemblages (with 17, 12 and 21 OTUs for each assemblage) (Supplementary Table S4 and Supplementary Figure S6). 17/18 indicator OTUs identified for farmland and alpine tundra groups belong to the same Kendall’s group (corrected *p*-value <0.05). These results are consistent with the beta diversity pattern and indicates that agricultural practices in highland barley farmland and soil degradation in alpine tundra might have consistency in their effects on soil microbial communities.

### 3.4 Environmental factors shaping the beta diversity of bacterial communities

**Figure 4:**
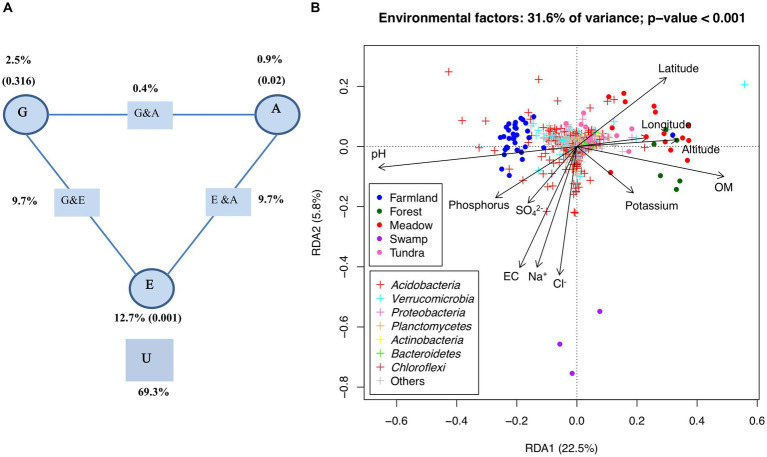
Deterministic factors shaping the bacterial communities. **(A)** Variation partition analysis of OTUs among geographic (“G”), elevation (“A”), edaphic attributes (“E”), and their interactions (“G x A,” “G x E,” and “E x A”). U, Unexplained variation (residual). The values in parentheses are *p*-values. **(B)** RDA of OTUs and environmental attributes. The percentage of variation explained by RDA1 and RDA2 is shown, both of the eigenvalues (RDA1 and RDA2) and all of the environmental variables are significant in permutation tests (*α* = 0.05). OM, organic matter; phosphorus, available phosphorus; potassium, available potassium; EC, electrical conductivity.

RDA showed that 31.6% (based on adjusted R^2^_,_ *p*-value <0.001) of the variance of bacterial communities among tested samples could be explained by those selected environmental and spatial factors. Variation partitioning analysis (Figure 4A) further revealed that edaphic factors alone captured 12.7% of variance (*p*-value <0.001), while very limited variation could be attributed to pure effects of altitude (0.9%, *p*-value = 0.02) and geographic distance (2.5%, *p*-value = 0.316). By contrast, the combined effects of edaphic factors with either altitude (23.3%) or geographic distance (24.9%) apparently may play a greater role in shaping the observed beta diversity (Figure 4A). As shown in the RDA analysis (Figure 4B), pH and organic matter were the major environmental factors along the first axis (22.5% of total variance, *p*-value <0.001). The farmland cluster was separated from those of wild ecosystems, particularly meadow and forest, along these two edaphic factors. The meadow and forest samples were enriched with certain OTUs belonging to *Proteobacteria* and *Verrucomicrobia* while the farmland samples harbored more OTUs of *Acidobacteria* (Figure 4B). Significant correlations between some OTUs and other individual edaphic factors such as phosphorus, SO_4_^2−^, EC, Na^+^ and Cl^−^ were also observed (Figure 4B).

**Figure 5:**
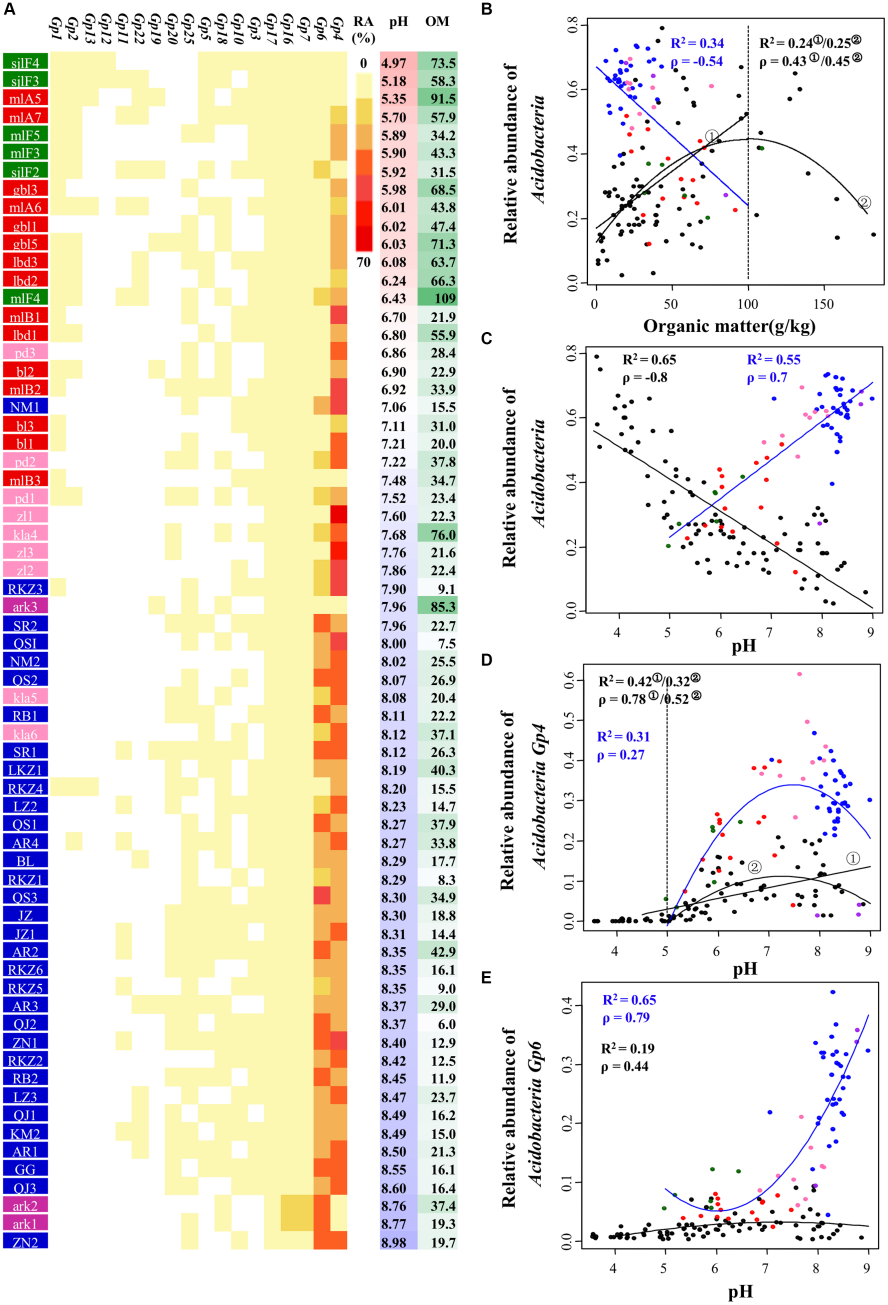
Relationship between the relative abundance of *Acidobacteria* and pH or organic matter in 66 soil bacterial communities. **(A)** The relative abundance of each *Acidobacteria* subgroup in each sample (ranging from 0 to 66.0%). OM, organic matter. **(B,C)** The effect of OM **(B)** or pH **(C)** on the abundance of *Acidobacteria*. **(D,E)** The effect of pH on the abundance of *Acidobacteria_Gp4* **(D)** and *Acidobacteria_Gp6* **(E)**. Samples were colored according to different ecosystems: farmland (blue), forest (green), meadow (red), swamp (purple), tundra (pink). Results from the research by Jones et al. (2009) were included in our analysis (black dots). Both Spearman’s rank correlation (ρ) and regression analysis (R^2^) were used for testing the correlations, all *p*-values <0.001.

*Acidobacteria* was the most abundant clade in tested samples. As shown in the heat map and scatterplots of the abundance of *Acidobacteria* subgroups corresponding to pH and organic matter (Figures 5A–E), relative abundance of *Acidobacteria* correlated negatively with soil organic matter (*R*^2^ = 0.34, Spearman *ρ* = −0.54, *p*-value <0.001) and positively with soil pH (*R*^2^ = 0.55, *ρ* = 0.70, *p*-value <0.001), i.e., higher in farmland and tundra soils and lower in the organic-rich acidic meadow and forest soils. This differs from the pattern uncovered from earlier reported data in North and South America (Jones et al., 2009) (Figures 5B,C). This discrepancy could be explained at the subgroup level, i.e., subgroups 1 (abundance range: 0 ~ 4.13%), 2 (0 ~ 5.20%), 3 (0 ~ 1.11%), 12 (0 ~ 0.05%), 13 (0 ~ 0.19%) and 15 (0%) showing negative correlations with pH were apparently less abundant than subgroups 4 (1.25 ~ 66.03%), 6 (2.46 ~ 43.28%), 7 (0.77 ~ 14.28%), 16 (0.27 ~ 12.62%), and 17 (0.10 ~ 5.33%) showing positive correlations with pH (Figures 5A,D,E).

In addition to the strong positive correlation with pH, relative abundance of *Acidobacteria* showed lower levels of correlation with EC (*ρ* = 0.414), available phosphorus (ρ = 0.365), Na^+^ (*ρ* = 0.297) and SO_4_^2−^ (*ρ* = 0.288) (all *p*-values <0.05). When these edaphic factors were individually controlled, the partial correlation coefficient of pH and relative abundance of *Acidobacteria* ranged from 0.65 to 0.8 (all *p*-values <0.001). If the other edaphic factors were all controlled, the coefficient was 0.41 (*p*-value = 0.0007). This implies that the positive correlation between pH and relative abundance of *Acidobacteria* was robust in our test samples. By contrast, the partial correlation coefficient was −0.17 (*p*-value >0.05) between organic matter and *Acidobacteria* abundance when the other edaphic factors were all controlled, indicating a combined effect by organic matter and other edaphic attributes.

## 4 Discussion

Understanding patterns and processes of bacterial biogeography is an important way to evaluate the ecological effect caused by human activities (Geremia et al., 2016; Wu et al., 2021; Xue et al., 2021). Soil biodiversity is strongly influenced by external drivers such as climate change and nitrogen deposition but also by land-use management (Wall et al., 2015). Evidence is mounting that soil biodiversity loss and simplification of soil community composition impair multiple ecosystem functions, including plant diversity, decomposition, nutrient retention, and nutrient cycling (Wagg et al., 2014). The Qinghai-Xizang Plateau, an ecologically vulnerable region, has undergone studies investigating soil bacterial communities in individual wild ecosystems like alpine meadows and lake sediments (Xiong et al., 2012; Yang et al., 2014; Yuan et al., 2014; Wang et al., 2022; Yang et al., 2022). In the present study, we compared soil bacterial communities in the highland barley farmland to those of wild alpine ecosystems including tundra, meadow, forest and swamp along the Yarlung Zangbo River valley, which is the major region for highland barley cultivation.

Soil bacterial communities were highly homogeneous in the highland barley farmland compared to those of meadow, forest and swamp. The observed low beta diversity in farmland samples might be partially due to the relatively simple vegetation coverage by highland barley compared to that of meadow, forest and swamp. This view is supported by a report of soil microbial communities along four sites of a Qinghai-Xizang mountainous grassland (Yang et al., 2014), where vegetation diversity was revealed as one of the major factors shaping the variations of microbial communities. Although farmland samples showed a similar level of beta diversity to those from tundra, no significant relationship was found between the beta diversity of farmland samples and their corresponding geographic, elevation and edaphic distances. This is in contrast to the observations in meadow samples in this study and earlier reports on bacterial communities in lake sediments and alpine meadows in the Qinghai-Xizang Plateau (Xiong et al., 2012; Yang et al., 2014; Yuan et al., 2014). It might be reasonable to speculate that transport via human activities, such as those in the farmland, could be more efficient than dispersal via wind, water, and other hitchhiking mechanisms in wild ecosystems (Papke and Ward, 2004). Recent studies demonstrated that land conversion to agricultural land results in taxonomic and functional homogenization of soil bacteria and fungi, mainly driven by the increase in the geographic ranges of taxa in croplands (Peng et al., 2024) and disproportionate negative effects of agricultural cultivation on rare microbial taxa (Banerjee et al., 2024). These results align with our perspective, indicating that agricultural activities lead to microbial homogenization and has profound implications for the structure and function of soil ecosystems, highlighting the importance of agricultural sustainability in protecting biodiversity.

The dominant phylum was *Acidobacteria* in all ecosystems tested in this study, and this is in line with earlier studies of soil bacterial communities across continents suggesting *Acidobacteria* as ubiquitous and abundant members (Zinger et al., 2009; Yuan et al., 2014; Sun et al., 2020; Sui et al., 2022). Moreover, farmland, tundra and swamp soils characterized with higher pH and lower organic matter than meadow and forest soils had a higher abundance of *Acidobacteria*. This is consistent with a negative correlation between abundance of *Acidobacteria* and organic carbon availability, and a strong positive correlation between pH and *Acidobacteria* abundance. These results are inconsistent with an earlier report in North and South America soils (Jones et al., 2009) but could be partially explained at the subgroup level of *Acidobacteria*, since subgroups such as 1, 2, 3, 12 and 13 which have negative correlations with pH (Jones et al., 2009) were less abundant in test samples of this study than those subgroups (4, 6, 7, 16, and 17) that have positive correlations with pH. Therefore, the general conclusion of the correlation between *Acidobacteria* abundance and pH/organic carbon availability proposed earlier (Fierer et al., 2007; Jones et al., 2009) might be misleading. These findings suggest that more studies should be compared to improve our understanding of the ecological significance of *Acidobacteria* (Kielak et al., 2016), and it is important to investigate bacterial communities at a high-resolution level (Liao et al., 2023).

Agricultural intensification can reduce the diversity and density of beneficial organisms; less-intensive practices such as enhancing crop diversity by rotation and intercropping, and reducing soil tillage are beneficial to soil biodiversity and the sustainability of agriculture (Wall et al., 2015; Bender et al., 2016). In this study, a considerable similarity level of bacterial communities between farmland and tundra soils was found. Tundra is characterized by its lower vegetation coverage than meadow and forest, and represents one of the most vulnerable ecosystems on the planet (Malanson et al., 2009; Sistla and Schimel, 2013; Stark et al., 2015). Future action should be taken to increase the crop diversity of barley farmland system by using rotation and intercropping crops (Bardgett and van der Putten, 2014; Bender et al., 2016; Blaser et al., 2016). Moreover, similar edaphic conditions (such as pH and organic matter) between the highland barley farmland and tundra suggest that the nutrient management of highland barley farmland soils should be carried out. Particularly pH and organic matter representing the edaphic factors with major effects on soil bacterial community could be managed in agriculture practices (van Rijssel et al., 2022; Wang et al., 2023). While this study provides valuable insights into the bacterial biogeography of highland barley farmland and wild alpine ecosystems, it has some limitations. Firstly, our study focused on the taxonomic biogeography of bacterial communities alone, a more comprehensive analysis incorporating multiple microbial groups (e.g., bacteria, fungi and archaea) and microbial function could provide a broader perspective on the effects of land-use change. Secondly, here we focused on a relatively small geographical scale in the Qinghai-Xizang Plateau, limiting the generalizability of the findings. Broader spatial sampling across different ecoregions and elevations could provide a more comprehensive understanding of bacterial biogeography patterns in alpine ecosystems.

## 5 Conclusion

An important aim of studies of soil bacterial community is to improve our understanding of the effects of human activities on the sustainability of agricultural soils. Our study highlights the distinctiveness of soil bacterial communities in highland barley farmland compared to those in wild alpine ecosystems along the Yarlung Zangbo River valley. The homogeneity observed in farmland bacterial communities, despite their similarity to tundra samples, but with no detectable correlations with abiotic effectors, suggests that agricultural activities in highland barley farmland may be having a profound effect on soil microbial diversity. This finding raises concerns about the potential ecological consequences of agricultural practices in this region, especially given the already fragile nature of the Qinghai-Xizang Plateau.

## Data availability statement

The datasets presented in this study can be found in online repositories. The names of the repository/repositories and accession number(s) can be found in the article/Supplementary material.

## Author contributions

XW: Visualization, Validation, Investigation, Funding acquisition, Conceptualization, Writing – review & editing, Writing – original draft, Supervision, Resources, Project administration, Methodology, Formal analysis, Data curation. YY: Writing – review & editing, Formal analysis. QN: Writing – review & editing. J-WG: Writing – review & editing. ZT: Writing – review & editing. XS: Supervision, Investigation, Funding acquisition, Conceptualization, Writing – review & editing. CT: Writing – original draft, Conceptualization, Writing – review & editing, Validation, Supervision, Resources, Funding acquisition.

## Footnotes

^1^ http://www.diva-gis.org/

## References

[ref1] Banerjee S. Zhao C. Garland G. Edlinger A. García-Palacios P. Romdhane S.. (2024). Biotic homogenization, lower soil fungal diversity and fewer rare taxa in arable soils across Europe. Nat. Commun. 15:327. doi: 10.1038/s41467-023-44073-6, PMID: 38184663 PMC10771452

[ref2] Bardgett R. D. van der Putten W. H. (2014). Belowground biodiversity and ecosystem functioning. Nature 515, 505–511. doi: 10.1038/nature13855 25428498

[ref3] Bender S. F. Wagg C. van der Heijden M. G. (2016). An underground revolution: biodiversity and soil ecological engineering for agricultural sustainability. Trends Ecol. Evol. 31, 440–452. doi: 10.1016/j.tree.2016.02.016, PMID: 26993667

[ref4] Blaser M. J. Cardon Z. G. Cho M. K. Dangl J. L. Donohue T. J. Green J. L.. (2016). Toward a predictive understanding of earth's microbiomes to address 21st century challenges. MBio 7:16. doi: 10.1128/mBio.00714-16, PMID: 27178263 PMC4895116

[ref5] Bokulich N. A. Subramanian S. Faith J. J. Gevers D. Gordon J. I. Knight R.. (2013). Quality-filtering vastly improves diversity estimates from Illumina amplicon sequencing. Nat. Methods 10, 57–59. doi: 10.1038/nmeth.2276, PMID: 23202435 PMC3531572

[ref6] Cao H. Chen R. Wang L. Jiang L. Yang F. Zheng S.. (2016). Soil pH, total phosphorus, climate and distance are the major factors influencing microbial activity at a regional spatial scale. Sci. Rep. 6:25815. doi: 10.1038/srep25815, PMID: 27170469 PMC4864422

[ref7] Caporaso J. G. Lauber C. L. Walters W. A. Berg-Lyons D. Huntley J. Fierer N.. (2012). Ultra-high-throughput microbial community analysis on the Illumina HiSeq and MiSeq platforms. ISME J. 6, 1621–1624. doi: 10.1038/ismej.2012.8, PMID: 22402401 PMC3400413

[ref8] Clarke K. R. (1993). Nonparametric multivariate analyses of changes in community structure. Aust. J. Ecol. 18, 117–143. doi: 10.1111/j.1442-9993.1993.tb00438.x

[ref9] Delgado-Baquerizo M. Oliverio A. M. Brewer T. E. Benavent-González A. Eldridge D. J. Bardgett R. D.. (2018). A global atlas of the dominant bacteria found in soil. Science 359, 320–325. doi: 10.1126/science.aap9516, PMID: 29348236

[ref10] Dini-Andreote F. Stegen J. C. van Elsas J. D. Salles J. F. (2015). Disentangling mechanisms that mediate the balance between stochastic and deterministic processes in microbial succession. Proc. Natl. Acad. Sci. USA 112, E1326–E1332. doi: 10.1073/pnas.1414261112, PMID: 25733885 PMC4371938

[ref11] Dufrene M. Legendre P. (1997). Species assemblages and indicator species: the need for a flexible asymmetrical approach. Ecol. Monogr. 67, 345–366. doi: 10.1890/0012-9615(1997)067[0345:SAAIST]2.0.CO;2

[ref12] Edgar R. C. Haas B. J. Clemente J. C. Quince C. Knight R. (2011). UCHIME improves sensitivity and speed of chimera detection. Bioinformatics 27, 2194–2200. doi: 10.1093/bioinformatics/btr381, PMID: 21700674 PMC3150044

[ref13] Feng L. Zhang Z. Yang G. Wu G. Yang Q. Chen Q. (2023). Microbial communities and sediment nitrogen cycle in a coastal eutrophic lake with salinity and nutrients shifted by seawater intrusion. Environ. Res. 225:115590. doi: 10.1016/j.envres.2023.115590, PMID: 36863651

[ref14] Fierer N. Bradford M. A. Jackson R. B. (2007). Toward an ecological classification of soil bacteria. Ecology 88, 1354–1364. doi: 10.1890/05-1839 17601128

[ref15] Fierer N. Jackson R. B. (2006). The diversity and biogeography of soil bacterial communities. Proc. Natl. Acad. Sci. USA 103, 626–631. doi: 10.1073/pnas.0507535103, PMID: 16407148 PMC1334650

[ref16] Geremia R. A. Puscas M. Zinger L. Bonneville J. M. Choler P. (2016). Contrasting microbial biogeographical patterns between anthropogenic subalpine grasslands and natural alpine grasslands. New Phytol. 209, 1196–1207. doi: 10.1111/nph.13690, PMID: 26443332

[ref17] Hanson C. A. Fuhrman J. A. Horner-Devine M. C. Martiny J. B. H. (2012). Beyond biogeographic patterns: processes shaping the microbial landscape. Nat. Rev. Microbiol. 10, 497–506. doi: 10.1038/nrmicro2795 22580365

[ref18] Hawkins J. H. Zeglin L. H. (2022). Microbial dispersal, including Bison dung vectored dispersal, increases soil microbial diversity in a grassland ecosystem. Front. Microbiol. 13:825193. doi: 10.3389/fmicb.2022.825193, PMID: 35432281 PMC9009311

[ref19] Helmus M. R. Bland T. J. Williams C. K. Ives A. R. (2007). Phylogenetic measures of biodiversity. Am. Nat. 169, E68–E83. doi: 10.1086/511334 17230400

[ref20] Jiao S. Chu H. Zhang B. Wei X. Chen W. Wei G. (2022). Linking soil fungi to bacterial community assembly in arid ecosystems. iMeta 1:e2. doi: 10.1002/imt2.2 38867731 PMC10989902

[ref21] Jones C. T. Meynell L. Neto C. Susko E. Bielawski J. P. (2023). The role of the ecological scaffold in the origin and maintenance of whole-group trait altruism in microbial populations. BMC Ecol. Evol. 23:11. doi: 10.1186/s12862-023-02112-2, PMID: 37046187 PMC10100367

[ref22] Jones R. T. Robeson M. S. Lauber C. L. Hamady M. Knight R. Fierer N. (2009). A comprehensive survey of soil acidobacterial diversity using pyrosequencing and clone library analyses. ISME J. 3, 442–453. doi: 10.1038/ismej.2008.127, PMID: 19129864 PMC2997719

[ref23] Kielak A. M. Barreto C. C. Kowalchuk G. A. van Veen J. A. Kuramae E. E. (2016). The ecology of Acidobacteria: moving beyond genes and genomes. Front. Microbiol. 7:744. doi: 10.3389/fmicb.2016.00744, PMID: 27303369 PMC4885859

[ref24] Kozich J. J. Westcott S. L. Baxter N. T. Highlander S. K. Schloss P. D. (2013). Development of a dual-index sequencing strategy and curation pipeline for analyzing amplicon sequence data on the MiSeq Illumina sequencing platform. Appl. Environ. Microbiol. 79, 5112–5120. doi: 10.1128/AEM.01043-13, PMID: 23793624 PMC3753973

[ref25] Kumar M. Brader G. Sessitsch A. Maki A. van Elsas J. D. Nissinen R. (2017). Plants assemble species specific bacterial communities from common core taxa in three Arcto-alpine climate zones. Front. Microbiol. 8:12. doi: 10.3389/fmicb.2017.00012 28174556 PMC5258723

[ref26] Larin A. K. Klimina K. M. Veselovsky V. A. Olekhnovich E. I. Morozov M. D. Boldyreva D. I.. (2024). An improved and extended dual-index multiplexed 16S rRNA sequencing for the Illumina HiSeq and MiSeq platform. BMC Genom. Data 25:8. doi: 10.1186/s12863-024-01192-3, PMID: 38254005 PMC10804484

[ref27] Legendre P. (2005). Species associations: the Kendall coefficient of concordance revisited. J. Agric. Biol. Environ. Stat. 10, 226–245. doi: 10.1198/108571105X46642

[ref28] Legendre P. Legendre L. (2012). Numerical ecology (developments in environmental modelling). second Edn. New York: Elsevier.

[ref29] Liao H. Ji Y. Sun Y. (2023). High-resolution strain-level microbiome composition analysis from short reads. Microbiome 11:183. doi: 10.1186/s40168-023-01615-w, PMID: 37587527 PMC10433603

[ref30] Malanson G. P. Brown D. G. Butler D. R. Cairns D. M. Fagre D. B. Walsh S. J. (2009). “Chapter 3 ecotone dynamics: Invasibility of alpine tundra by tree species from the subalpine forest,” in Developments in earth surface processes. eds. Butler D. R. Malanson G. P. Walsh S. J. Fagre D. B. (Elsevier), 35–61.

[ref31] Nemergut D. R. Schmidt S. K. Fukami T. O'Neill S. P. Bilinski T. M. Stanish L. F.. (2013). Patterns and processes of microbial community assembly. Microbiol. Mol. Biol. Rev. 77, 342–356. doi: 10.1128/MMBR.00051-12, PMID: 24006468 PMC3811611

[ref32] Oksanen J. Blanchet F. G. Friendly M. Kindt R. Legendre P. McGlinn D.. (2016). Vegan: Community ecology package. R package version 2.4–1. Available at: https://CRAN.R-project.org/package=vegan

[ref33] Papke R. T. Ward D. M. (2004). The importance of physical isolation to microbial diversification. FEMS Microbiol. Ecol. 48, 293–303. doi: 10.1016/j.femsec.2004.03.013, PMID: 19712299

[ref34] Parks D. H. Porter M. Churcher S. Wang S. Blouin C. Whalley J.. (2009). GenGIS: a geospatial information system for genomic data. Genome Res. 19, 1896–1904. doi: 10.1101/gr.095612.109, PMID: 19635847 PMC2765287

[ref35] Peng Z. Qian X. Liu Y. Li X. Gao H. An Y.. (2024). Land conversion to agriculture induces taxonomic homogenization of soil microbial communities globally. Nat. Commun. 15:3624. doi: 10.1038/s41467-024-47348-8, PMID: 38684659 PMC11058813

[ref36] Price M. N. Dehal P. S. Arkin A. P. (2009). FastTree: computing large minimum evolution trees with profiles instead of a distance matrix. Mol. Biol. Evol. 26, 1641–1650. doi: 10.1093/molbev/msp077, PMID: 19377059 PMC2693737

[ref37] Ruff S. E. Biddle J. F. Teske A. P. Knittel K. Boetius A. Ramette A. (2015). Global dispersion and local diversification of the methane seep microbiome. Proc. Natl. Acad. Sci. USA 112, 4015–4020. doi: 10.1073/pnas.1421865112, PMID: 25775520 PMC4386351

[ref38] Schloss P. D. Westcott S. L. Ryabin T. Hall J. R. Hartmann M. Hollister E. B.. (2009). Introducing mothur: open-source, platform-independent, community-supported software for describing and comparing microbial communities. Appl. Environ. Microbiol. 75, 7537–7541. doi: 10.1128/AEM.01541-09, PMID: 19801464 PMC2786419

[ref39] Segata N. Izard J. Waldron L. Gevers D. Miropolsky L. Garrett W. S.. (2011). Metagenomic biomarker discovery and explanation. Genome Biol. 12:R60. doi: 10.1186/gb-2011-12-6-r60, PMID: 21702898 PMC3218848

[ref40] Shapiro B. J. Friedman J. Cordero O. X. Preheim S. P. Timberlake S. C. Szabo G.. (2012). Population genomics of early events in the ecological differentiation of bacteria. Science 336, 48–51. doi: 10.1126/science.1218198, PMID: 22491847 PMC3337212

[ref41] Simpson E. H. (1949). Measurement of diversity. Nature 163:688. doi: 10.1038/163688a0

[ref42] Sistla S. A. Schimel J. P. (2013). Seasonal patterns of microbial extracellular enzyme activities in an arctic tundra soil: identifying direct and indirect effects of long-term summer warming. Soil Biol. Biochem. 66, 119–129. doi: 10.1016/j.soilbio.2013.07.003

[ref43] Stark S. Männistö M. K. Ganzert L. Tiirola M. Häggblom M. M. (2015). Grazing intensity in subarctic tundra affects the temperature adaptation of soil microbial communities. Soil Biol. Biochem. 84, 147–157. doi: 10.1016/j.soilbio.2015.02.023

[ref44] Stewart C. N. Excoffier L. (1996). Assessing population genetic structure and variability with RAPD data: application to *Vaccinium macrocarpon* (American cranberry). J. Evol. Biol. 9, 153–171. doi: 10.1046/j.1420-9101.1996.9020153.x

[ref45] Sui X. Frey B. Yang L. Liu Y. Zhang R. Ni H.. (2022). Soil Acidobacterial community composition changes sensitively with wetland degradation in northeastern of China. Front. Microbiol. 13:1052161. doi: 10.3389/fmicb.2022.1052161, PMID: 36620014 PMC9816132

[ref46] Sun H. Wu Y. Zhou J. Bing H. Zhu H. (2020). Climate influences the alpine soil bacterial communities by regulating the vegetation and the soil properties along an altitudinal gradient in SW China. Catena 195:104727. doi: 10.1016/j.catena.2020.104727

[ref47] van Rijssel S. Q. Veen G. F. Koorneef G. J. Bakx-Schotman J. M. T. ten Hooven F. C. Geisen S.. (2022). Soil microbial diversity and community composition during conversion from conventional to organic agriculture. Mol. Ecol. 31, 4017–4030. doi: 10.1111/mec.16571, PMID: 35726521 PMC9545909

[ref48] Wagg C. Bender S. F. Widmer F. van der Heijden M. G. A. (2014). Soil biodiversity and soil community composition determine ecosystem multifunctionality. Proc. Natl. Acad. Sci. USA 111, 5266–5270. doi: 10.1073/pnas.1320054111, PMID: 24639507 PMC3986181

[ref49] Wall D. H. Nielsen U. N. Six J. (2015). Soil biodiversity and human health. Nature 528, 69–76. doi: 10.1038/nature15744 26595276

[ref50] Walsh E. A. Kirkpatrick J. B. Pockalny R. Sauvage J. Spivack A. J. Murray R. W.. (2016). Relationship of bacterial richness to organic degradation rate and sediment age in subseafloor sediment. Appl. Environ. Microbiol. 82, 4994–4999. doi: 10.1128/AEM.00809-16, PMID: 27287321 PMC4968545

[ref51] Wan W. Gadd G. M. Gu J. Liu W. Chen P. Zhang Q.. (2023). Beyond biogeographic patterns: processes shaping the microbial landscape in soils and sediments along the Yangtze River. mLife 2, 89–100. doi: 10.1002/mlf2.12062, PMID: 38818339 PMC10989888

[ref52] Wang X. L. Cui W. J. Feng X. Y. Zhong Z. M. Li Y. Chen W. X.. (2018). Rhizobia inhabiting nodules and rhizosphere soils of alfalfa: a strong selection of facultative microsymbionts. Soil Biol. Biochem. 116, 340–350. doi: 10.1016/j.soilbio.2017.10.033

[ref53] Wang S. Jiao C. Zhao D. Zeng J. Xing P. Liu Y.. (2022). Disentangling the assembly mechanisms of bacterial communities in a transition zone between the alpine steppe and alpine meadow ecosystems on the Tibetan plateau. Sci. Total Environ. 847:157446. doi: 10.1016/j.scitotenv.2022.157446, PMID: 35863578

[ref54] Wang C. Yu Q.-Y. Ji N.-N. Zheng Y. Taylor J. W. Guo L.-D.. (2023). Bacterial genome size and gene functional diversity negatively correlate with taxonomic diversity along a pH gradient. Nat. Commun. 14:7437. doi: 10.1038/s41467-023-43297-w, PMID: 37978289 PMC10656551

[ref55] Wu H. Hao B. Cai Y. Liu G. Xing W. (2021). Effects of submerged vegetation on sediment nitrogen-cycling bacterial communities in Honghu Lake (China). Sci. Total Environ. 755:142541. doi: 10.1016/j.scitotenv.2020.142541, PMID: 33039889

[ref56] Xiong J. Liu Y. Lin X. Zhang H. Zeng J. Hou J.. (2012). Geographic distance and pH drive bacterial distribution in alkaline lake sediments across Tibetan plateau. Environ. Microbiol. 14, 2457–2466. doi: 10.1111/j.1462-2920.2012.02799.x, PMID: 22676420 PMC3477592

[ref57] Xu X. Wang N. Lipson D. Sinsabaugh R. Schimel J. He L.. (2020). Microbial macroecology: in search of mechanisms governing microbial biogeographic patterns. Glob. Ecol. Biogeogr. 29, 1870–1886. doi: 10.1111/geb.13162

[ref58] Xue R. Zhao K. Yu X. Stirling E. Liu S. Ye S.. (2021). Deciphering sample size effect on microbial biogeographic patterns and community assembly processes at centimeter scale. Soil Biol. Biochem. 156:108218. doi: 10.1016/j.soilbio.2021.108218

[ref59] Yang Y. Gao Y. Wang S. Xu D. Yu H. Wu L.. (2014). The microbial gene diversity along an elevation gradient of the Tibetan grassland. ISME J. 8, 430–440. doi: 10.1038/ismej.2013.146 23985745 PMC3906809

[ref60] Yang F. Ma C. Fang H. (2022). Simulation of critical transitions and vulnerability assessment of Tibetan plateau key ecosystems. J. Mt. Sci. 19, 673–688. doi: 10.1007/s11629-021-6960-7

[ref61] Yuan Y. Si G. Wang J. Luo T. Zhang G. (2014). Bacterial community in alpine grasslands along an altitudinal gradient on the Tibetan plateau. FEMS Microbiol. Ecol. 87, 121–132. doi: 10.1111/1574-6941.12197, PMID: 23991911

[ref62] Zeng X. Long H. Wang Z. Zhao S. Tang Y. Huang Z.. (2015). The draft genome of Xizang Highland barley reveals adaptive patterns to the high stressful Tibetan plateau. Proc. Natl. Acad. Sci. USA 112, 1095–1100. doi: 10.1073/pnas.1423628112, PMID: 25583503 PMC4313863

[ref63] Zhang H. Gao S. Lercher M. J. Hu S. Chen W. H. (2012). EvolView, an online tool for visualizing, annotating and managing phylogenetic trees. Nucleic Acids Res. 40, W569–W572. doi: 10.1093/nar/gks576, PMID: 22695796 PMC3394307

[ref64] Zinger L. Shahnavaz B. Baptist F. Geremia R. A. Choler P. (2009). Microbial diversity in alpine tundra soils correlates with snow cover dynamics. ISME J. 3, 850–859. doi: 10.1038/ismej.2009.20, PMID: 19322246

