# Supplementary material for: Barley farmland harbors a highly homogeneous soil bacterial community compared to wild ecosystems in the Qinghai-Xizang Plateau
Source: Front Microbiol. 2024 Jun 24;15:1418161. doi: 10.3389/fmicb.2024.1418161 (PMC11228161; doi:10.3389/fmicb.2024.1418161)
Supplement: Supplementary file 2 [file Data_Sheet_2.PDF]

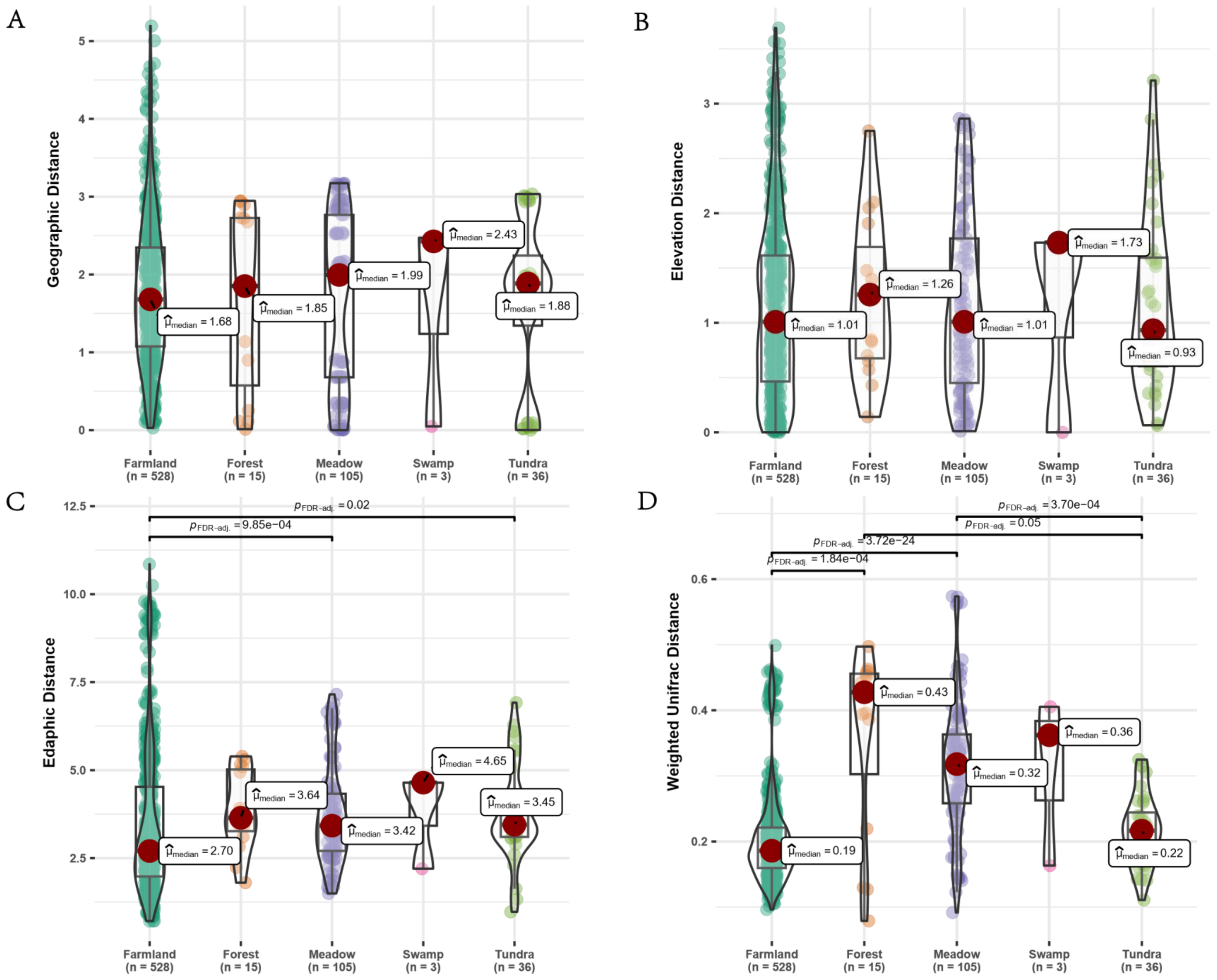

Figure S1. Pairwise comparisons of the distribution of within-group distances based on the Euclidean distances of geographic (A), elevation (B) or edaphic characteristics (C), as well as the weighted UniFrac distance of soil bacterial communities (D). A combination of box and violin plots along with jittered data points for each group, FDR adjusted p-values (using Benjamini-Hochberg adjustment) were used for multiple comparisons after nonparametric Dunn's test, an alpha-value of 0.05 was chosen as our significance cutoff and with bars shown in the plots.

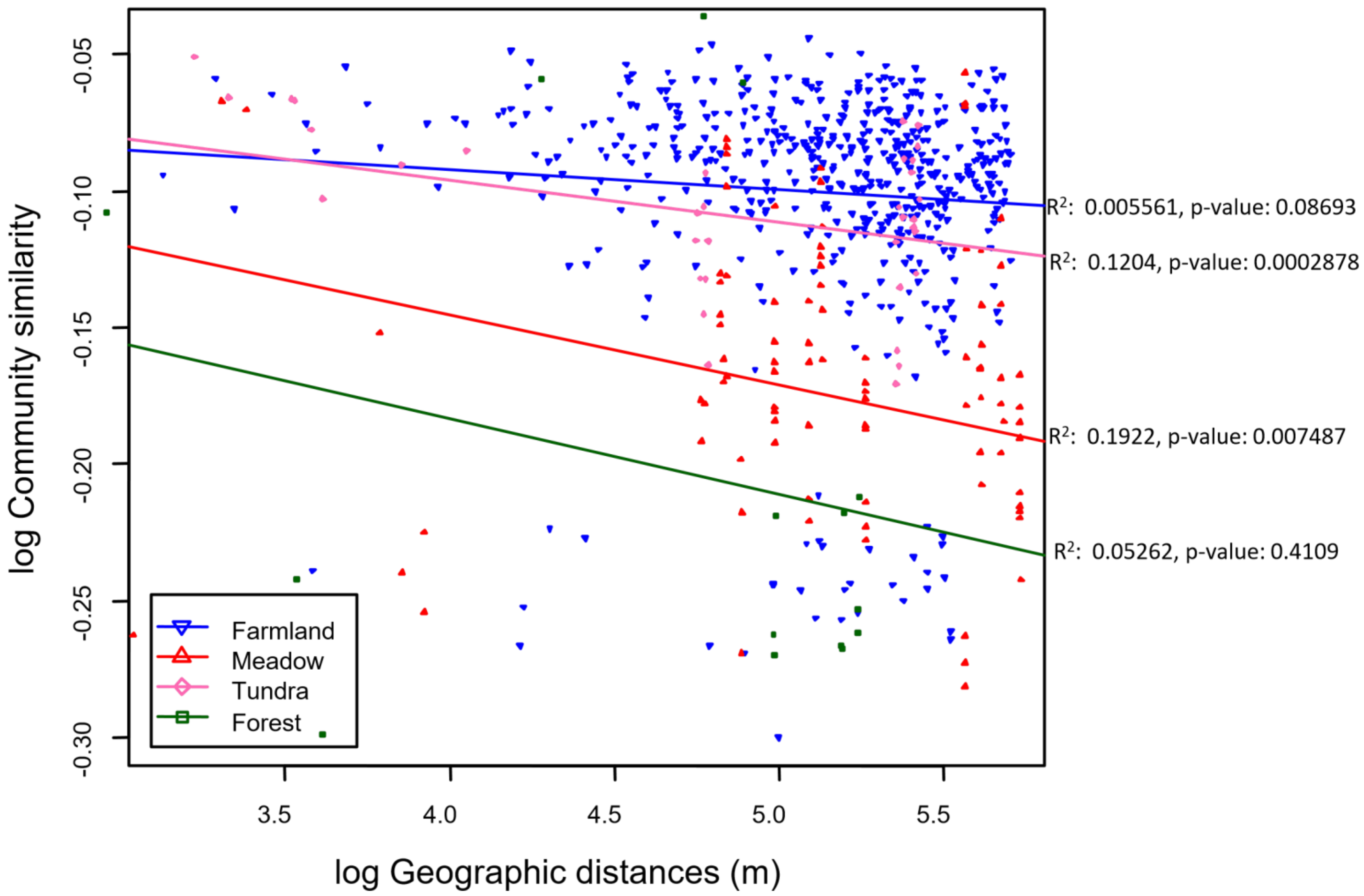

Figure S2. Distance decay analysis. The community similarity used here represents one minus bacterial weighted UniFrac distance. The colored lines denote the least-squares linear regression for each ecosystem, with R^2 and p-value on the right of each line.

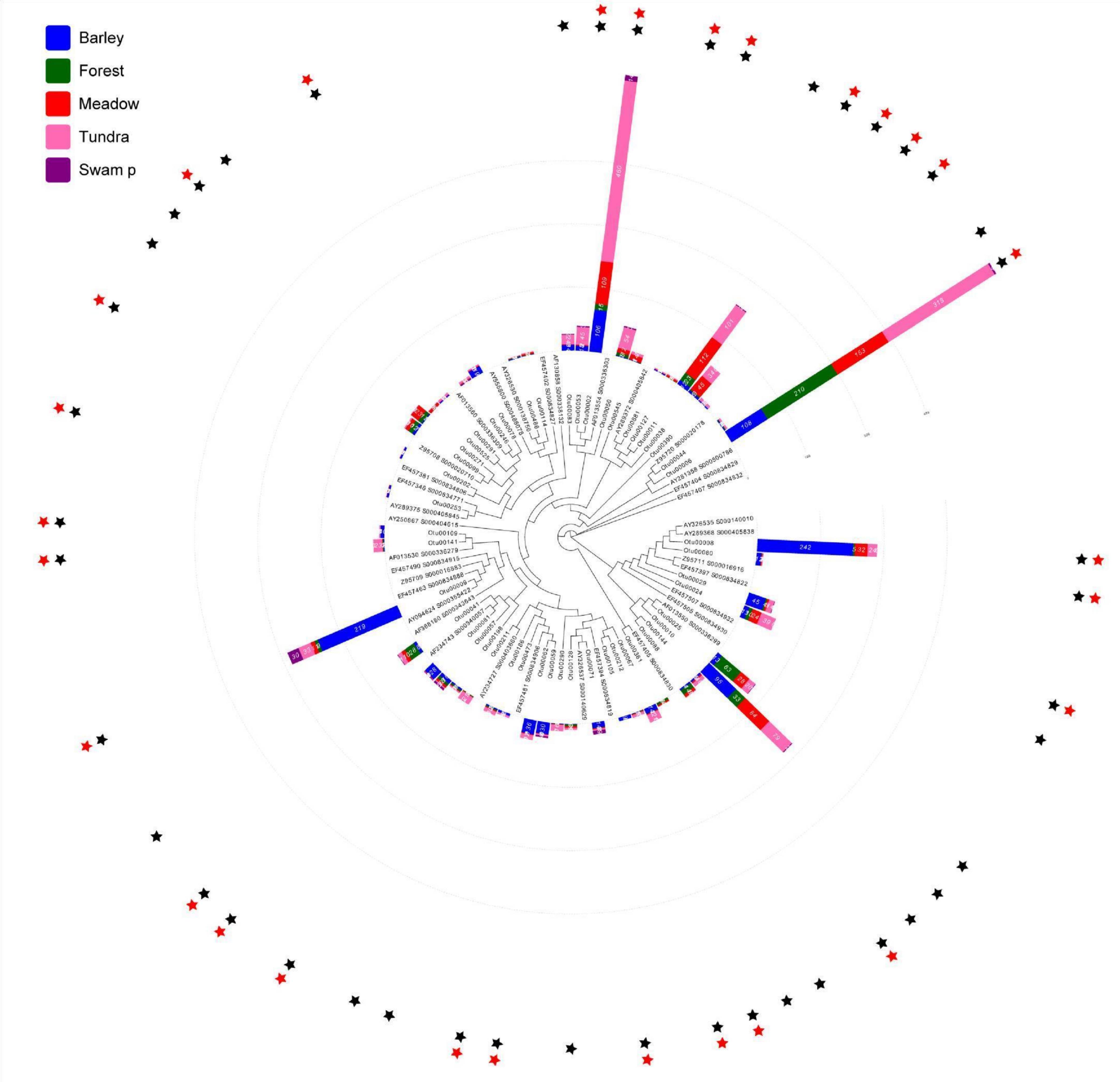

Figure S3. Phylogenetic tree of *Acidobacteria Gp4* and its relative abundance between Xizang ecosystems. Only representative OTUs with more than 100 reads in the total samples were visualized (49 sequences), with the reference *Acidobacteria Gp4* sequences extracted from the SILVA database. Black star: OTUs with significant different abundance among five ecosystems (Kruskal Wallis test, p-value < 0.05); red star, OTUs with significant different abundance between THB farmland and alpine tundra samples (Kruskal Wallis test, p-value < 0.05).

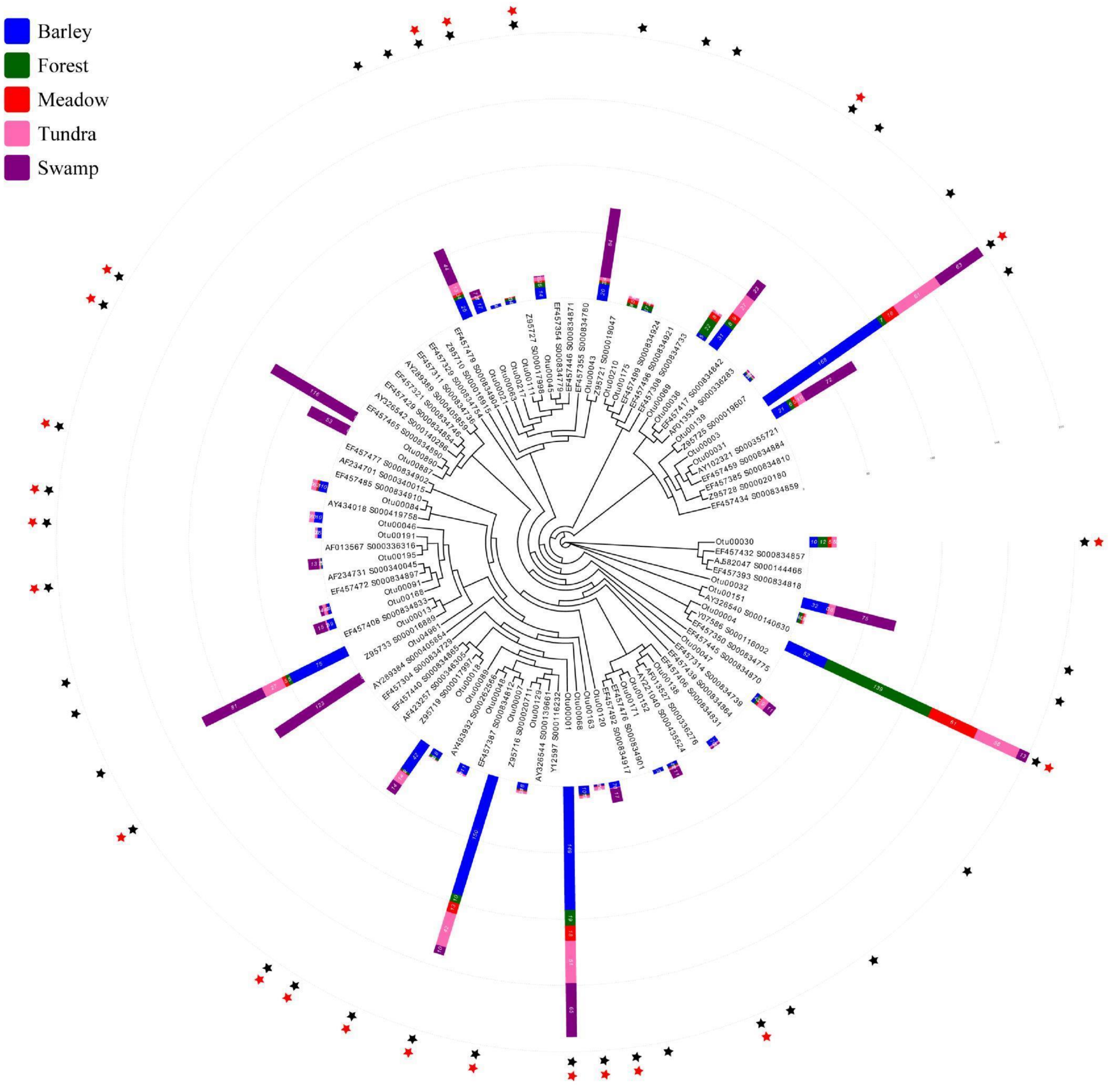

Figure S4. Phylogenetic tree of *Acidobacteria Gp6* and its relative abundance between Xizang ecosystems. Only representative OTUs with more than 100 reads in the total samples were visualized (40 sequences), with the reference *Acidobacteria Gp6* sequences extracted from the SILVA database. Black star: OTUs with significant different abundance among five ecosystems (Kruskal Wallis test, p-value < 0.05); red star, OTUs with significant different abundance between THB farmland and swamp samples (Kruskal Wallis test, p-value < 0.05).

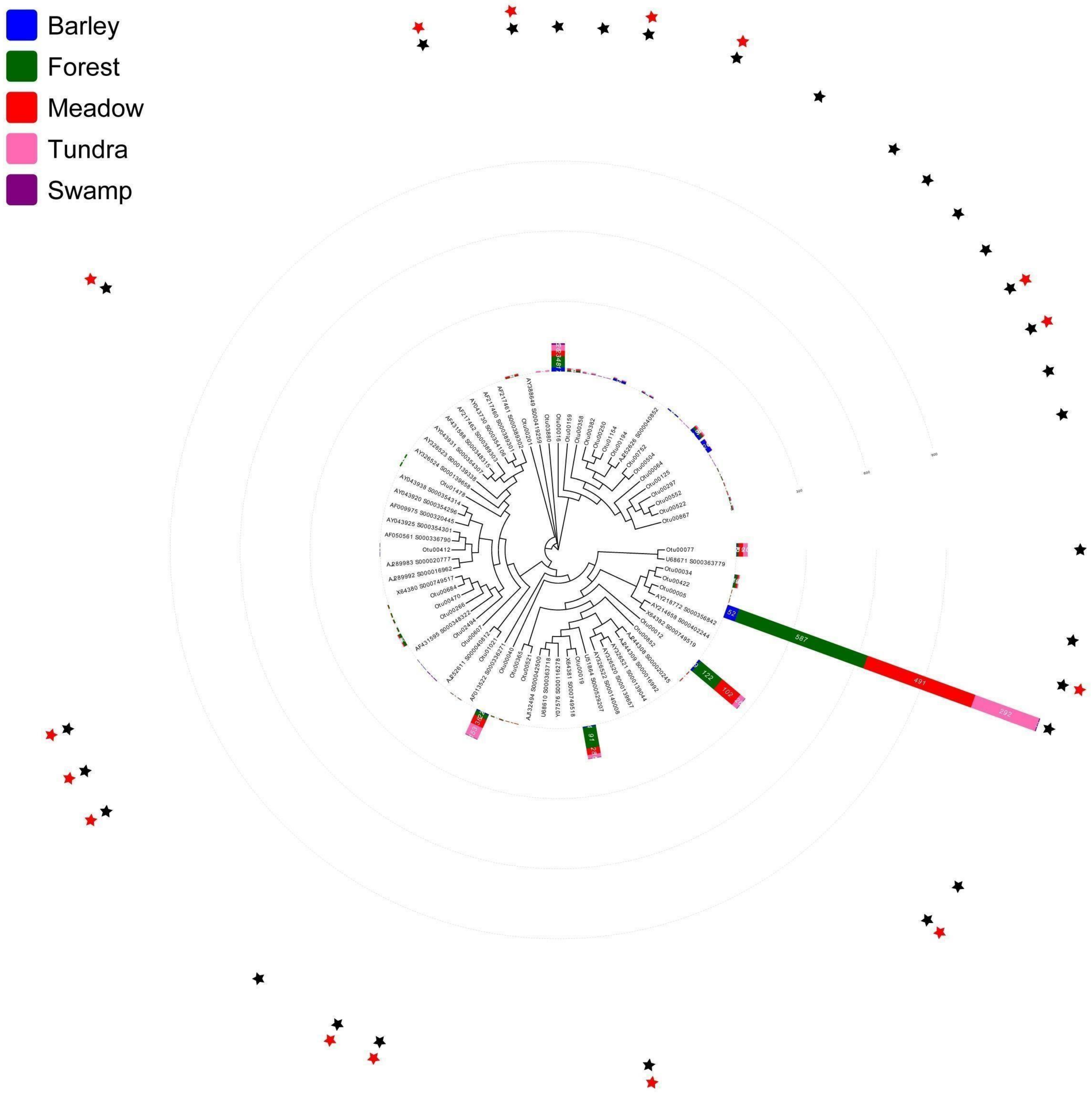

Figure S5. Phylogenetic tree of *Spartobacteria* and its relative abundance between Xizang ecosystems. Only representative OTUs with more than 20 reads in the total samples were visualized (35 sequences), with the reference *Spartobacteria* sequences extracted from the SILVA database. Black star: OTUs with significantly different abundance among five ecosystems (Kruskal Wallis test, p-value < 0.05); red star, OTUs with significantly different abundance among forest, meadow and tundra samples (Kruskal Wallis test, p-value < 0.05).

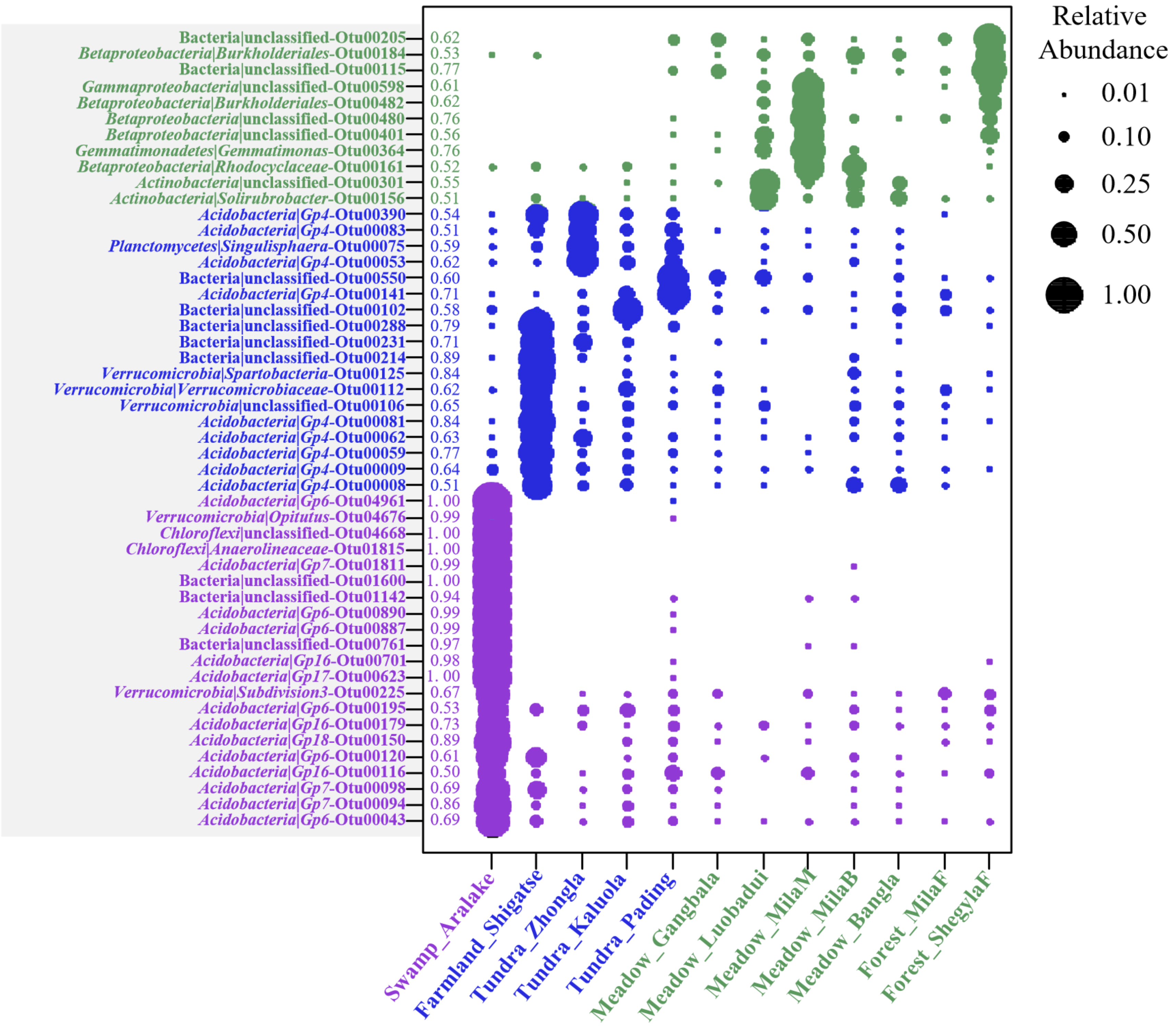

Figure S6. Indicator species and species assemblages analysis based on high abundant OTUs. Considering that the bacterial community of farmland soils was highly homogeneous and its beta diversity was not correlated to tested environmental factors (Figure 1B and Table 2), a group of three samples from Shigatse county was used to represent farmland samples in the analysis of indicator OTUs for ensure the consistency of sample number. On the other hand, all the 33 non-farmland samples of 11 groups defined by their geographic origins were used.OTUs representing larger than 0.05% of total reads were used for indicator species analysis. 50 OTUs with indicate value > 0.5 and p-value < 0.05 for the twelve groups were plotted, with indicator value on the right of the OTUs' labels. The average relative abundances of each indicator OTU among all groups were normalized (i.e., each line sums to 1), and the area of the bubbles were pictured according to the proportion of each OTU among groups. The bubbles were colored according to the species assemblages identified using Kendall's W coefficient of concordance, purple for indicator OTUs belonging to swamp, blue for for indicator OTUs of farmland and tundra, and green for indicator OTUs of forest and meadow.
